# Terlipressin Treatment for Acute Esophageal Variceal Bleeding: Bolus or Infusion?

**DOI:** 10.5152/tjg.2025.25265

**Published:** 2025-08-25

**Authors:** Ali Şenkaya, Ferit Çelik, İlkçe Kurtulmuş Akgün, Seymur Aslanov, Alper Uysal, Abdullah Murat Buyruk, Nalan Gülşen Ünal, İlker Turan, Ulus Salih Akarca, Zeki Karasu

**Affiliations:** 1 Gastroenterology Outpatient Clinic, İzmir City Hospital, İzmir, Türkiye; 2 Gastroenterology Outpatient Clinic, İzmir Çiğli Training and Research Hospital, İzmir, Türkiye; 3 Department of Internal Medicine, Ege University Faculty of Medicine, izmir, Türkiye; 4 Gastroenterology Outpatient Clinic, İzmir Medicalpoint Hospital, İzmir, Türkiye; 5 Gastroenterology Outpatient Clinic, İstanbul Kanuni Sultan Süleyman Training and Research Hospital, İstanbul, Türkiye; 6 Department of Internal Medicine, Division of Gastroenterology, Ege University Faculty of Medicine, İzmir, Türkiye

**Keywords:** Acute variceal bleeding, cirrhosis, mortality, portal hypertension, terlipressin, vasoactive agents

## Abstract

**Background/Aims:**

This study aimed to compare the efficacy of bolus versus infusion administration of terlipressin in patients with acute esophageal variceal bleeding and to elucidate any differences in clinical outcomes between the 2 approaches.

**Materials and Methods:**

This prospective study included patients divided into 2 groups. Group 1 received a 2 mg intravenous (IV) bolus followed by 1 mg IV every 4 hours. Group 2 received a 1 mg IV bolus followed by a 4 mg terlipressin infusion over 24 hours. Clinical and laboratory parameters, hospitalization duration, need for blood product transfusion, rebleeding or mortality within 6 weeks, and drug-related side effects were evaluated.

**Results:**

Among the 46 patients, 23 (50%) received terlipressin as an IV bolus (group 1), and 23 (50%) received it as an infusion (group 2). Treatment failure occurred in 4 patients (8.7%), all from group 1, though the difference was not statistically significant (*P* = .109). Six patients (13%) experienced rebleeding and death within 6 weeks, with no significant differences in clinical outcomes between the groups. No significant differences in creatinine and sodium levels were observed between the groups at baseline or at the end of treatment (*P* = .654). Additionally, no difference in the incidence of portal vein thrombosis was noted between survivors and non-survivors (*P* = 1.000).

**Conclusion:**

As no significant differences in efficacy or safety were observed between bolus and infusion administration, infusion therapy may be preferred due to its potential benefits in patient comfort and ease of administration.

**Main Points**

- There were no significant differences in creatinine and sodium levels between the bolus (group 1) and infusion (group 2) groups at the beginning or end of treatment.
- Bolus and infusion terlipressin administration demonstrated comparable efficacy, mortality, rebleeding rates, side effects, and treatment discontinuation within 6 weeks.
- Infusion therapy may be a suitable and cost-effective alternative, with potential for reduced dosing frequency and improved patient comfort.

## Introduction

Esophageal variceal bleeding is a common and life-threatening complication of portal hypertension, affecting nearly half of all patients with cirrhosis.^1^ Annually, 5%-15% of cirrhotic patients experience variceal bleeding, with reported mortality rates ranging from 7% to 20%.^2-4^ Standard treatment strategies include pharmacological agents such as vasoactive drugs, endoscopic interventions like sclerotherapy and endoscopic band ligation (EBL), and advanced modalities such as balloon tamponade, transjugular intrahepatic portosystemic shunt, or surgery.^5-7^ A combination of endoscopic and pharmacologic therapy is considered the most effective approach.^6^

Among endoscopic treatments, EBL is the most effective for acute esophageal variceal bleeding. The primary objective of endoscopic therapy is to reduce variceal wall tension and eradicate varices; however, it does not directly affect portal pressure.^8^ In contrast, medical management targets a reduction in splanchnic blood flow and portal pressure to control acute hemorrhage from esophageal varices.^9^ Vasoactive agents such as vasopressin, terlipressin, somatostatin, and octreotide are commonly employed to achieve hemostasis and prevent rebleeding.^2,9-11^

Terlipressin, a synthetic analog of vasopressin, exhibits potent vasoconstrictive activity with a relatively favorable side effect profile. Its mechanism of action involves the activation of V1 receptors located primarily on the smooth muscle of splanchnic arteries, leading to reduced splanchnic blood flow and portal pressure, thereby effectively controlling acute variceal hemorrhage.^12,13^ Terlipressin is typically administered at doses of 1-2 mg intravenously every 4 hours, although some protocols recommend a 2 mg dose every 6 hours.^5^ According to the Baveno VI consensus, terlipressin treatment should not exceed 5 consecutive days.^4^ Recent research has also demonstrated that continuous infusion of terlipressin can significantly lower portal pressure.^14,15^

Although both bolus and infusion routes are recommended for terlipressin administration in acute esophageal variceal bleeding, direct comparative studies remain limited. At the institution, terlipressin is administered using both approaches. Therefore, the present prospective study aimed to comparatively evaluate the efficacy and safety of bolus versus infusion methods of terlipressin administration in this clinical setting.

## Materials and Methods

This prospective study included patients with acute esophageal variceal bleeding who underwent EBL and received terlipressin treatment in the Gastroenterology Department of Ege University hospital between January and December 2020. The diagnosis of portal hypertension was based on clinical, biochemical, ultrasonographic, and/or histopathological findings. The inclusion and exclusion criteria are summarized in Table 1, and patient data were recorded in case report forms as detailed in Table 2.

Following hemodynamic stabilization, upper gastrointestinal endoscopy was performed within 12 hours of hospital admission. Treatment failure was defined as the occurrence of 100 mL or more of fresh hematemesis within 2 hours of EBL, a decrease in hemoglobin of ≥3 g/dL without transfusion, or death. Terlipressin was administered in a sequential pattern, with 1 patient receiving bolus therapy and the next receiving infusion therapy. Based on the mode of administration, patients were divided into 2 groups. Group 1 received 2 mg of terlipressin as an intravenous (IV) bolus followed by 1 mg IV every 4 hours. Group 2 received a 1 mg IV bolus followed by a continuous infusion of 4 mg terlipressin over 24 hours.

The study was approved by the local ethics committee of Ege University (Approval date: December 25, 2019; Approval No. 19-12.1T/20) and conducted in accordance with the principles of the Declaration of Helsinki.All patients provided informed consent.

### Statistical Analysis

All statistical analyses were performed using SPSS software version 22 (IBM SPSS Corp.; Armonk, NY, USA). Descriptive statistics are expressed as mean ± standard deviation (SD) for numerical variables and as frequencies and percentages for categorical variables. The Kolmogorov–Smirnov test was used to assess the normality of distribution. For comparisons between groups, the student’s *t*-test was used for normally distributed numerical variables, while the chi-square test or Fisher’s exact test was applied for categorical variables. Friedman’s test was employed to assess changes in laboratory parameters over time. When necessary, pairwise comparisons were conducted using the Wilcoxon signed-rank test, with Bonferroni correction applied. A 2-sided *P* value of <.05 was considered statistically significant.

## Results

**Figure 1.**
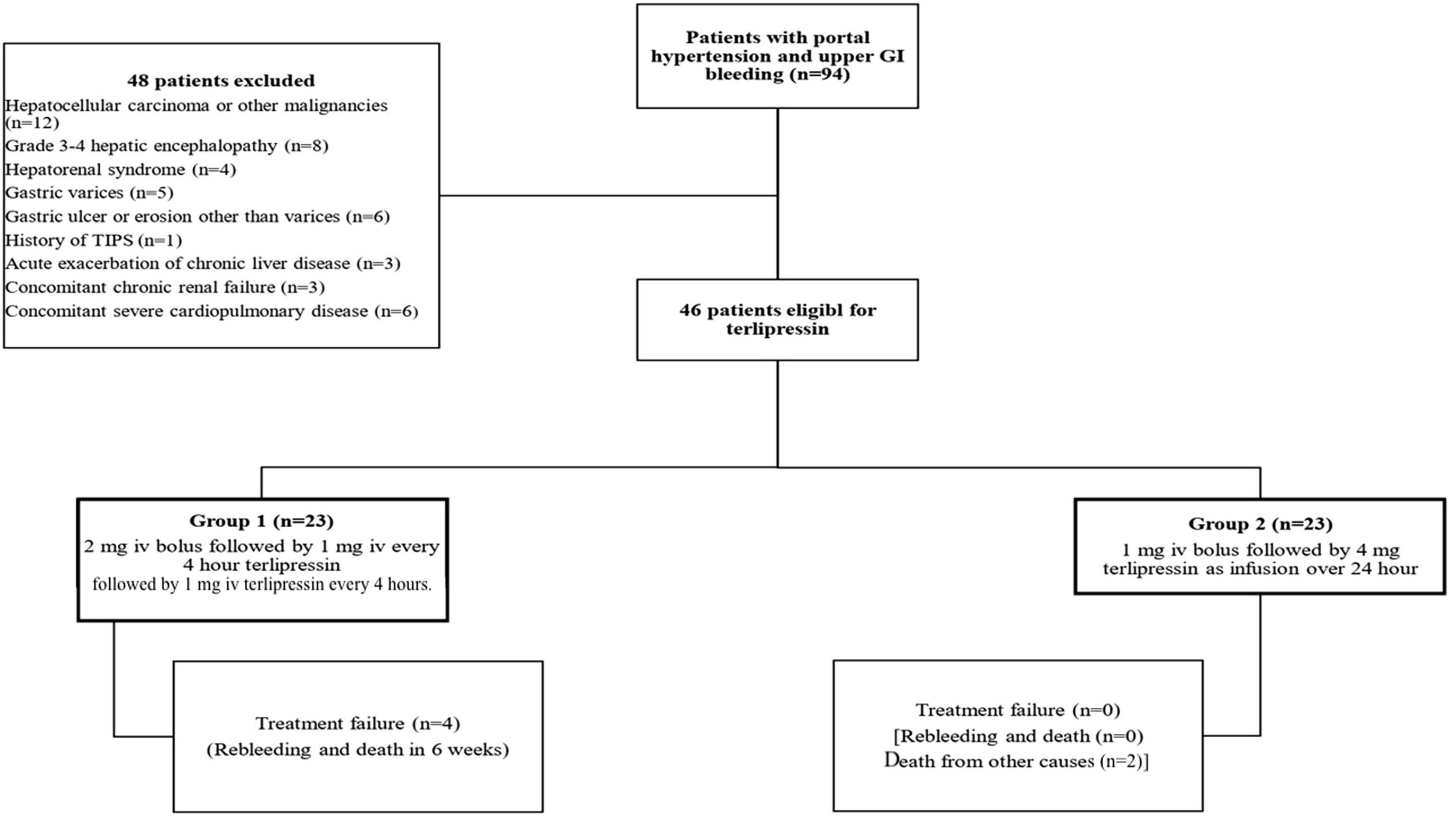
Flowchart of patient selection and study design. TIPS, transjugular intrahepatic portosystemic shunt.

The study flowchart is presented in Figure 1. Of the 46 patients included, 23 (50%) received terlipressin as an IV bolus (group 1), and 23 (50%) received it as an IV infusion (group 2). Overall, 29 patients (63%) were male, and the mean age was 58.7 ± 12.7 years. A history of variceal bleeding was noted in 20 patients (43.5%), hepatic encephalopathy in 10 (21.7%), and prior EBL in 27 (58.7%). There were no statistically significant differences between groups regarding these variables (*P* = 1.000, *P* = .475, and *P* = .369, respectively).

Regarding medication history, 3 patients (6.5%) had used nonsteroidal anti-inflammatory drugs, 2 (4.3%) were on antiaggregant therapy, 4 (8.7%) were receiving anticoagulants, 20 (43.5%) were on diuretics, and 24 (52.2%) were taking prophylactic beta-blockers. There were no significant differences in medication use between the 2 groups.

At hospital admission, 16 patients (34.8%) presented with grade 1-2 hepatic encephalopathy, and 24 (52.2%) had nonrefractory mild ascites. Again, there were no significant intergroup differences (*P* = .536 and *P* = .555, respectively). However, the number of comorbidities differed significantly between the groups (*P* = .041). The demographic and clinical characteristics of the patients, along with their comparison by treatment type, are summarized in Table 3.

Laboratory and ultrasonographic findings are presented in Table 4. No significant differences were found between treatment groups regarding these parameters (*P* > .05). Similarly, endoscopic outcomes and treatment characteristics did not differ significantly between the 2 groups (Table 5).

The mean hospital stay was 9.8 ± 5.9 days. Treatment failure occurred in 4 patients (8.7%), all in group 1, although this difference was not statistically significant compared with group 2 (*P* = .109). Six patients experienced rebleeding and died within 6 weeks. Of these, 1 patient in group 2 died within the first 24 hours due to acute kidney injury and respiratory failure, and another in group 2 died due to acute-on-chronic liver failure (ACLF). The remaining 4 patients, all from group 1, died due to rebleeding and treatment failure. No statistically significant differences in clinical outcomes were found between treatment groups (Table 6).

There were no significant differences in serum creatinine or sodium levels between groups 1 and 2 at the beginning or end of treatment (*P* = .654).

During terlipressin therapy, electrocardiographic changes were observed in 4 patients—3 from group 1 and 1 from group 2. These changes included T-wave inversions in the anterior leads (n = 2), ST-segment depression (n = 1), and moderate sinus tachycardia (n = 1). None of these findings necessitated treatment discontinuation. All affected patients continued therapy following cardiology consultation.

## Discussion

In this study comparing the efficacy of bolus versus infusion terlipressin treatment in patients with acute esophageal variceal bleeding, no significant differences were observed between the 2 groups regarding drug-related side effects, length of hospital stay, need for blood product transfusion, mortality, or rebleeding. These findings contribute meaningfully to the existing body of literature on terlipressin administration strategies.

Terlipressin has a half-life of approximately 50 minutes and cannot maintain therapeutic plasma levels beyond 4 hours.^16^ This pharmacokinetic limitation has led to investigations comparing bolus with continuous infusion regimens. Due to its potential for serious adverse effects—such as myocardial ischemia—terlipressin has also been used at lower doses via infusion in patients with septic shock to mitigate such risks.^17^ Moreover, low-dose infusion of terlipressin has been shown to be effective in the treatment of hepatorenal syndrome and is associated with fewer side effects than bolus administration.^18^

When administering terlipressin, it is important to monitor oxygen saturation (ensuring levels >90%) as well as signs of ischemia, arrhythmias, and blood pressure changes. Daily electrocardiography (ECG), along with monitoring of blood pressure, pulse, and oxygen saturation, are recommended. Regular evaluation of serum creatinine, sodium, and potassium levels is also essential.

In the present study, no significant differences were observed between the 2 treatment groups in terms of side effects requiring treatment discontinuation. Although ECG changes were more frequently observed in group 1, this difference was not statistically significant. These findings are consistent with those of Jha et al,^15^ who also reported no difference in adverse event profiles. In contrast, a recent study by Arora et al^19^ reported a significantly higher rate of side effects in the bolus group than the infusion group (bolus: 56.4% vs. infusion: 36.3%, *P* = .03). Similarly, Vaishnav et al^20^ compared 1- and 3-day bolus terlipressin regimens and found that shorter treatment duration was associated with fewer side effects (37.8% vs. 56%, *P* = .026). Although bolus administration is theoretically expected to be associated with more adverse events, this was not confirmed in the study.

The study found no significant differences between the bolus and infusion groups in terms of treatment efficacy, mortality, or rebleeding at 6 weeks. However, earlier studies by Jha and Arora^15,19^ reported higher rates of early rebleeding and mortality in the bolus group. In this study, 4 of the 6 total deaths occurred in group 1 and were attributed to treatment failure and rebleeding, whereas the 2 deaths in group 2 were due to respiratory failure, acute kidney injury, and ACLF. Although not statistically significant, the number of treatment failures (4 vs. 2; *P* = .109) and the number of patients with rebleeding and death within 6 weeks (4 vs. 2; *P* = .665) were higher in the bolus group, potentially supporting findings from previous studies.^15,19^

Amitrano et al^21^ suggested that the presence of portal vein thrombosis is an unfavorable prognostic factor in acute variceal bleeding. In this study, although 6 patients in group 2 had portal vein thrombosis, the number of deaths was lower in this group than in group 1, and this difference was not statistically significant. Interestingly, despite a higher burden of comorbidities in group 2, mortality was numerically lower than in group 1.

This study has several limitations. The relatively small sample size and the inability to measure hepatic venous pressure gradients limit the generalizability of the findings. Additionally, the low incidence of treatment failure and mortality restricted the statistical power to identify prognostic factors. Despite these limitations, the findings align with current evidence suggesting comparable efficacy between bolus and infusion administration, with the potential added benefits of lower drug doses, fewer side effects, and cost-effectiveness in the infusion group.

In conclusion, this study found no statistically significant differences between bolus and infusion terlipressin treatment in terms of treatment efficacy, mortality, rebleeding within 6 weeks, side effects, or treatment discontinuation. Although not statistically significant, the infusion group exhibited fewer adverse events. Based on these findings and the potential for reduced drug use and cost, terlipressin infusion therapy may be considered in the management of acute esophageal variceal bleeding.

## Figures and Tables

**Table 1.** Inclusion and Exclusion Criteria of the Study

Inclusion Criteria	Exclusion Criteria
Patients presenting with hematemesis and/or melena, with symptoms occurring within 24 hours prior to enrollment	Hepatocellular carcinoma or other malignancies
Presence of active bleeding in esophageal varices on endoscopy, a clot on an esophageal varix, or varices in patients with portal hypertension with no other bleeding source in the upper gastrointestinal tract	Grade 3-4 hepatic encephalopathy
	Hepatorenal syndrome
	Gastric varices
	Gastric ulcer or erosion other than varices
	History of TIPS
	HIV
	Acute exacerbation of chronic liver disease
	Concomitant chronic renal failure
	Concomitant severe cardiopulmonary disease
	Patients under 18 years of age

HIV, human immunodeficiency virus; TIPS, transjugular intrahepatic portosystemic shunt.

**Table 2.** Data Collection Parameters Used in the Study

Data Category	Details
Demographic data	Age, gender, etc.
Etiology of portal hypertension	–
History of esophageal variceal bleeding	–
History of endoscopic band ligation	–
History of hepatic encephalopathy	–
Medications used	Prophylactic beta-blockers, diuretics, antiaggregant/anticoagulant agents, and NSAIDs
Comorbidities	–
Child–Pugh score (on admission)	–
MELD and MELD-Na score (on admission)	–
Time to endoscopy after bleeding	–
Endoscopic findings	Active bleeding, red color sign, degree of varices, number of bands applied
Laboratory values	Leukocyte, hemoglobin, hematocrit, platelet, INR, albumin, AST, ALT, ALP, GGT, total bilirubin, urea, creatinine, and sodium
Portal Doppler USG findings	Portal vein diameter, presence of portal vein thrombosis, spleen size
Duration of hospitalization	–
Blood product transfusion (during hospitalization)	Erythrocyte suspension, fresh frozen plasma
Rebleeding within 6 weeks	–
Death within 6 weeks	–
Admission ECG	–
Daily ECG findings	–
Terlipressin-associated side effects	–
Time to onset of side effect	–
Duration of side effect	–
Nature of side effect (if any)	–
Whether treatment was discontinued	–

ALP, alkaline phosphatase; ALT, alanine aminotransferase; AST, aspartate aminotransferase; ECG, electrocardiography; GGT, gamma-glutamyl transferase; INR, international normalized ratio; MELD, model for end-stage liver disease; MELD-Na, model for end-stage liver disease-sodium; NSAID, nonsteroidal anti-inflammatory drug; USG, ultrasonography.

**Table 3.** Comparison of Demographic and Clinical Characteristics of the Patients by Terlipressin Treatment Type

	All PatientsMean ± SD	Type of Terlipressin Treatment		*P*
		Group 1Mean ± SD	Group 2Mean ± SD	
Age (years)	58.7 ± 12.7	55.6 ± 15.1	61.7 ± 9.2	.110
Child–Pugh score	8.1 ± 2.27	8.04 ± 2.27	8.2 ± 2.32	.848
MELD-Na	14.28 ± 5.95	14.91 ± 6.04	13.65 ± 5.92	.479
Sex, n (%) Female Male	17 (37)29 (63)	8 (34.8)15 (65.2)	9 (39.1)14 (60.9)	.760
Comorbidity, n (%) None One disease Two or more diseases	13 (28.3)19 (41.3)14 (30.4)	10 (43.5)9 (39.1)4 (17.4)	3 (13)10 (43.5)10 (43.5)	.041^*^
Etiology of portal hypertension, n (%) Cryptogenic Alcohol HBV NASH HCV HBV + HDV Cardiac PVT Others^a^	14 (30.4)9 (19.6)7 (15.2)5 (10.9)2 (4.3)2 (4.3)2 (4.3)2 (4.3)3 (6.6)	6 (26.1)5 (21.7)5 (21.7)1 (4.3)1 (4.3)1 (4.3)1 (4.3)1 (4.3)2 (8.6)	8 (34.8)4 (17.4)2 (8.7)4 (17.4)1 (4.3)1 (4.3)1 (4.3)1 (4.3)1 (4.3)	.647
Child–Pugh classification, n (%) A B C	15 (32.6)17 (37)14 (30.4)	7 (30.4)10 (43.5)6 (26.1)	8 (34.8)7 (30.4)8 (34.8)	.643

HBV, hepatitis B virus; HCV, hepatitis C virus; HDV, hepatitis D virus; MELD-Na, model for end-stage liver disease-sodium; NASH, non-alcoholic steatohepatitis; PVT, portal vein thrombosis; SD, standard deviation.

^a^Autoimmune hepatitis (n = 1), Wilson’s disease (n = 1), Budd–Chiari syndrome (n = 1).

**Table 4.** Baseline Laboratory and Ultrasonography Results of the Patients by Terlipressin Treatment Type

	All PatientsMean ± SD	Type of Terlipressin Treatment		*P*
		Group 1Mean ± SD	Group 2Mean ± SD	
Laboratory values				
Leukocyte	7.636.1 ± 4.168	6.995 ± 4.700	8.277 ± 3.547	.302
Hemoglobin	8.05 ± 2.21	8.08 ± 2.2	8.01 ± 2.27	.911
Hematocrit (%)	25.3 ± 6.14	25.04 ± 6.12	25.5 ± 6.28	.799
Platelets	108 587 ± 5.7750	99 391 ± 60.526.3	117 782.6 ± 54.601	.285
INR	1.47 ± 0.38	1.54 ± 0.4	1.4 ± 0.3	.203
AST	41.15 ± 30	34.2 ± 20.4	48.1 ± 36.4	.116
ALT	28.5 ± 26.2	22.7 ± 16.3	34.3 ± 32.6	.133
ALP	112.3 ± 86	99.7 ± 53.1	125 ± 109.4	.324
GGT	82.8 ± 85.7	63.1 ± 56.8	102.6 ± 104.8	.119
Total bilirubin	2.06 ± 2.45	2.51 ± 3.28	1.6 ± 1.02	.213
Albumin	29.77 ± 5.92	29.1 ± 6.4	30.4 ± 5.4	.461
Urea	54.17 ± 26.75	49.1 ± 24.9	59.3 ± 28.1	.201
Creatinine	0.82 ± 0.32	0.77 ± 0.33	0.88 ± 0.32	.244
Sodium	135.44 ± 4.57	135.04 ± 5.16	135.83 ± 3.98	.568
Ultrasonography results				
Portal vein diameter (mm)	14.2 ± 2.38	14.3 ± 2.75	14.1 ± 1.99	.807
Spleen size (cm)	16.9 ± 1.84	17.1 ± 1.78	16.7 ± 1.91	.527
Portal vein thrombosis None, n (%) Present, n (%)	38 (82.6)8 (17.4)	21 (91.3)2 (8.7)	17 (73.9)6 (26.1)	.243

ALP, alkaline phosphatase; ALT, alanine aminotransferase; AST, aspartate aminotransferase; GGT, gamma-glutamyl transferase; INR, international normalized ratio; SD, standard deviation.

**Table 5.** Endoscopic and Treatment Characteristics by Terlipressin Administration Type

	All Patients	Type of Terlipressin Treatment		*P*
		Group 1	Group 2	
Endoscopy duration, hours (mean ± SD)	10.21 ± 7.51	10.13 ± 7.87	10.30 ± 7.30	.938
Number of EBLs (mean ± SD)	5.17 ± 1.52	5.13 ± 1.48	5.21 ± 1.59	.849
Active bleeding on endoscopy, n (%)	21 (45.7)	11 (47.8)	10 (43.5)	.767
Red color sign, n (%)	42 (91.3)	20 (87)	22 (95.7)	.608
White nipple sign, n (%)	41 (89.1)	22 (95.7)	19 (82.6)	.346

EBL, endoscopic band ligation; SD, standard deviation.

**Table 6.** Treatment Outcomes by Terlipressin Administration Type

	All PatientsMean ± SD	Type of Terlipressin Treatment		*P*
		Group 1Mean ± SD	Group 2Mean ± SD	
Length of hospital stay (days)	9.8 ± 5.9	9.48 ± 4.9	10.13 ± 6.9	.712
ES replacement (units)	2.4 ± 1.9	2.7 ± 2.3	2.1 ± 1.5	.325
FFP replacement (units)	0.46 ± 0.83	0.7 ± 0.97	0.22 ± 0.6	.052
	**n (%) **	**n (%) **	**n (%) **	
Treatment failure	4 (8.7)	4 (17.4)	0 (0)	.109
Death at 6 weeks	6 (13)	4 (17.4)	2 (8.7)	.665
Rebleeding at 6 weeks	6 (13)	4 (17.4)	2 (8.7)	.665
Terlipressin side effects	4 (8.7)	3 (13)	1 (4.3)	.608
Treatment discontinuation	4 (8.7)	3 (13)	1 (4.3)	.608
ECG changes	4 (8.7)	3 (13)	1 (4.3)	.608

ECG, electrocardiography; ES, erythrocyte suspension; FFP, fresh frozen plasma; SD, standard deviation.

## Data Availability

The data that support the findings of this study are available on request from the corresponding author.
